# Impact of removable dentures on oral health-related quality of life among elderly adults in Taiwan

**DOI:** 10.1186/1472-6831-15-1

**Published:** 2015-01-05

**Authors:** Yea-Yin Yen, Huey-Er Lee, Yi-Min Wu, Shou-Jen Lan, Wen-Chen Wang, Je-Kang Du, Shun-Te Huang, Kun-Jung Hsu

**Affiliations:** Department of Oral Hygiene, College of Dental Medicine, Kaohsiung Medical University, Kaohsiung, Taiwan; School of Dentistry, College of Dental Medicine, Kaohsiung Medical University, Kaohsiung, Taiwan; Division of Prosthodontics, Department of Dentistry, Kaohsiung Medical University Hospital, Kaohsiung, Taiwan; Division of Periodontics, Department of Dentistry, Kaohsiung Medical University Hospital, Kaohsiung, Taiwan; Department of Healthcare Administration, College of Health Science, Asia University, Taichung, Taiwan; Division of Oral Pathology, Department of Dentistry, Kaohsiung Medical University Hospital, Kaohsiung, Taiwan; Department of Dentistry, Kaohsiung Municipal Ta-Tung Hospital, Kaohsiung, Taiwan; Division of Pediatric Dentistry, Department of Dentistry, Kaohsiung Medical University Hospital, Kaohsiung, Taiwan; Division of Family Dentistry, Department of Dentistry, Kaohsiung Medical University Hospital, Kaohsiung, Taiwan; Department of Dentistry, Kaohsiung Municipal Ci-Jin Hospital, Kaohsiung, Taiwan

**Keywords:** Elderly, Removable dental prostheses, Oral health-related quality of life, Geriatric oral health assessment index, Denture satisfaction, Dental care service

## Abstract

**Background:**

Although the use of removable dentures can improve oral function and esthetics for elderly people, compared to those who do not wear removable dentures, those wearing removable dentures could have worse oral health related-quality of life (OHRQoL). Additional information is required to assess which factors related to denture wearing influence the OHRQoL of elderly individuals. The purpose of this study is to evaluate the association between denture wearing and OHRQoL in a sample of elderly individuals in Taiwan.

**Methods:**

The study population included 277 elderly people wearing removable dentures (mean age = 76.0 years). Using face-to-face interviews, we collected data on the participants’ socio-demographic characteristics, dental care service usage (regular dental checkups, treatment during toothache, dental visits in the last year), and factors related to denture wearing (perceived oral pain, perceived loose denture, perceived oral ulcer, perceived halitosis, perceived dry mouth, and perceived total denture satisfaction scores). OHRQoL was measured using the Taiwanese version of the Geriatric Oral Health Assessment Index (GOHAI-T). The location and number of remaining natural teeth and the type of denture were also recorded. Hierarchical multiple regression analysis was performed using GOHAI-T scores as the dependent variable.

**Results:**

All the predictors together accounted for 50% of the variance in GOHAI-T scores. Further, education level, number of natural teeth, denture status, perceived loose denture, perceived oral ulcer, and perceived total denture satisfaction scores had statistically significant influences on OHRQoL. When compared with other variables, factors related to denture wearing, especially perceived total denture satisfaction scores, had the greatest impact on GOHAI-T scores.

**Conclusions:**

Of the factors analyzed in this study, denture satisfaction was the strongest predictor of OHRQoL. This suggests that denture satisfaction is useful for assessing the effect of denture treatment on the OHRQoL of elderly individuals wearing removable dentures.

## Background

Because of the low fertility rate in Taiwan [1], the elderly population is rapidly increasing. By the end of 2026, people aged 65 years or over are expected to account for 20.1% of the total population of Taiwan [2], and, as such, there is a growing interest in helping the elderly to live a complete and healthy life, physically, mentally, and socially [3]. Oral health is an important part of well-being, and oral health-related quality of life (OHRQoL) should be emphasized when providing dental care to elderly people. One of the most commonly used measures of OHRQoL is the Geriatric Oral Health Assessment Index (GOHAI), which was developed specifically for use with older adults [4].

Many previous studies have shown that implant-supported dentures can substantially improve the wearer’s quality of life [5]; however, the higher cost of treatment with dental implants means that removable dentures continue to be widely used to replace missing teeth [6], especially in the elderly [7]. Unfortunately, individuals wearing a removable prosthesis can experience significant problems with regard to the social and emotional aspects of life, as compared to individuals with natural teeth [8]. It maybe difficult for some individuals to adapt to dentures, as wearing a removable prosthesis demands emotional and functional adjustments [9]. As such, elderly individuals wearing removable dentures may experience more OHRQoL impairments than do those who do not wear removable dentures.

In elderly people, OHRQoL is known to be associated with socioeconomic status [10–13], regular dental visits [14], subjective masticatory ability [15], and the number of remaining natural teeth [12, 14, 16–18]. Factors related to denture wearing that specifically affect the OHRQoL of elderly people wearing removable dentures include denture status [18–21], denture satisfaction [10, 19], perceived loose denture [11, 22, 23], presence of oral pain [24], presence of oral ulcer [13], perceived halitosis [25] and perceived dry mouth [17]. Kuo et al. [7] found that increased denture satisfaction was significantly related to an improvement in elderly patients’ OHRQoL. In addition, Komagamine et al. [22] showed a positive relationship between retention of the lower denture and OHRQoL, using the Oral Health Impact Profile (OHIP), in edentulous patients. Moreover, Ekanayake et al. [16] reported a significant association between halitosis and higher OHIP scores, indicating that people with halitosis had a worse OHRQoL than those who did not have halitosis.

In addition, a previous study indicated that elderly patients’ denture satisfaction is associated with health-related quality of life (HRQoL) [10]. However, Inoue et al. [26] showed that HRQoL in patients with removable dentures was mediated by OHRQoL. Moreover, Lee et al. [27] observed that perceived oral health status, measured by the OHIP, had a greater impact on HRQoL than did the clinical factors, as measured by the 36-Item Short Form (SF-36) Health Survey. Therefore, it is important to assess the predictors of OHRQoL to improve HRQoL.

A previous study showed that although denture wearing could improve oral function and esthetics related to quality of life, people wearing removable dentures had a significantly higher median OHIP score than did those who do not wear removable dentures, indicating a poorer OHRQoL in the former group [16]. Hence, additional information is required to determine which factors related to denture wearing influence the OHRQoL of elderly subjects. Improved understanding of the factors correlated with a better OHRQoL would help in clinical decision making to provide dental treatment appropriate for patients’ specific needs and concerns. Although the relationship between variables related to denture wearing and OHRQoL has been investigated in previous studies [19, 22], it is unclear which variable is the strongest predictor of OHRQoL. Therefore, in the current study, we aimed to address this gap in the literature. The research hypothesis was that denture satisfaction would be the strongest predictor of OHRQoL among the variables mentioned above that relate to wearing of removable dentures.

## Methods

### Participants

This was a cross-sectional study carried out from September 2009 to January 2010. A convenience sample of elderly subjects aged 65 years or over who wore removable dentures was recruited from the Kaohsiung City Government Senior Citizens’ Service. Subjects were excluded from the study for the following reasons: 1. did not complete the questionnaire; 2. did not eat the foods listed in the questionnaire due to religious beliefs, vegetarianism, or other personal reasons; 3. had three or more incorrect answers on the Short Portable Mental Status Questionnaire (SPMSQ), indicating mild to severe intellectual impairment [28].Nine subjects were excluded from the study, 5 of them were vegetarian, 3 did not complete their questionnaire and additional 1 subject was mild intellectual impairment.

In the present study, the SPMSQ was used to evaluate the participants’ ability to understand the questionnaire. The cognitive status of elderly individuals was defined as the number of wrong answers in the 10-question SPMSQ, whereby two or fewer incorrect answers indicate an intact cognitive status, three or four incorrect answers indicate mild intellectual impairment, and five or more incorrect answers indicate moderate to severe intellectual impairment.

Written informed consent was obtained from all subjects prior to data collection. Ethical approval was obtained from the Institutional Review Board at Chung-Ho Memorial Hospital, Kaohsiung Medical University (KMUH–IRB–980273).

### Questionnaire

Information obtained by questionnaires included socio-demographic characteristics (age, gender, education level, living alone or with others, and the ability to afford living expenses), factors related to denture wearing (presence of oral pain, perceived loose denture, presence of oral ulcer, perceived halitosis, perceived dry mouth, and satisfaction with removable dentures), dental health service usage (regular dental checkups, treatment during toothache, dental visits in the last year), and OHRQoL, (as measured using the GOHAI).

OHRQoL was measured using the GOHAI, which was originally developed for use in older adult populations [4]. The GOHAI instrument provides a score based on the answers to 12 questions associated with the following three domains of OHRQoL: physical function (PF), including eating, speech, and swallowing; psychosocial function (PSF), including worry or concern about oral health, dissatisfaction with appearance, self-consciousness about oral health, and avoidance of social contact because of oral problems; and pain or discomfort (PD), including the use of medication to relieve pain or discomfort in the mouth. Score ranges for the dimensions of physical function, psychosocial function, and pain or discomfort are4–20, 5–25, and 3–15, respectively.

The original English Version of the GOHAI was translated into Chinese for use with Taiwanese people (GOHAI-T; see Table 1). A panel of professional experts carried out and verified the translation and cross-cultural adaptation of the original English version. Responses to GOHAI-T items were assessed using a 5-point Likert scale, ranging from 1 = always to 5 = never. Total scores on the GOHAI-T were a summation of all individual scores obtained from the 12 items, with a higher score indicating a lower impact on OHRQoL (range of sum score = 12–60). Denture satisfaction was evaluated by a denture-satisfaction assessment (DSA). In previous studies in which the DSA was utilized, six questions were used to evaluate the level of subjects’ satisfaction with their removable dentures [29–31]. The questions were related to tasting, retention (including upper and lower dentures), esthetics, comfort (including upper and lower dentures), appearance, ability to speak, and mastication ability. Subjects’ satisfaction was recorded using a 5-point Likert scale, wherein the scores ranged from 1 (very unsatisfied) to 5 (very satisfied). The scores for questions concerning retention and comfort of the dentures were calculated as average scores of the upper and lower dentures. Subjects’ responses to the six questions are summed to obtain a total denture satisfaction score. The sum score can range from 6 to 30, with a higher score indicating more satisfaction with the dentures.

**Table 1:** **English and Taiwanese versions of the GOHAI**

English questions	Taiwanese questions
1. How often did you limit the kinds or amounts of food you eat because of problems with your teeth or dentures?	
2. How often did you have trouble biting or chewing any kinds of food, such as firm meat or apples?	
3. How often were you able to swallow comfortably?	
4. How often have your teeth or dentures prevented you from speaking the way you wanted?	
5. How often were you able to eat anything without feeling discomfort?	
6. How often did you limit contacts with people because of the condition of your teeth or dentures?	
7. How often were you pleased or happy with the looks of your teeth and gums, or dentures?	
8. How often did you use medication to relieve pain or discomfort from around your mouth?	
9. How often were you worried or concerned about the problems with your teeth, gums, or dentures?	
10. How often did you feel nervous or self-conscious because of problems with your teeth, gums, or dentures?	
11. How often did you feel uncomfortable eating in front of people because of problems with your teeth or dentures?	
12. How often were your teeth or gums sensitive to hot, cold, or sweets?	

The answers of questions were scored by 5=always(總是),  4=usually(經常), 3=occasionally(偶爾),  2=seldom(很少), and 1=never(從不). The scores were reversed in the 3rd, 7th and 12th questions.

### Dental examination

The dental examinations were carried out by one of the authors, a family dentistry specialist with 15 years of clinical experience, in accordance with the guidelines of the World Health Organization [32]. Intra-examiner reliability (kappa coefficient) was assessed in a sample of 14 subjects during the data collection. The kappa coefficient for intra-examiner agreement was 0.90, which indicates a high level of inter-examiner agreement.

Information was collected on the location, number, and type of natural teeth, and the kind of dentures. Teeth that were sound, decayed, filled, or filled but decayed were marked as natural teeth. Teeth with grade III mobility, retained roots, or extensive crown destruction (i.e., at least three-fourths of the clinical crown destroyed) were excluded.

### Statistical analysis

We explored the relationships among the variables using STATA version 13.0 (Stata Corp, College Station, Texas USA), with the significance level set at 5%. Continuous variables and categorical variables are expressed as mean ± standard error (SE), counts (n), and percentages (%), respectively.

Bivariate analysis was used to compare GOHAI-T scores with socio-demographic characteristics, dentition and denture status, dental care service usage, and factors related to denture wearing. A t-test and one-way analysis of variance were used to assess the distributions of GOHAI-T scores in relation to the categorical variables. Correlations between the GOHAI-T scores and continuous variables were evaluated using Spearman’s rank correlation analysis.

The reliability of the GOHAI-T scale was evaluated using the internal consistency approach (Cronbach’s alpha) in the presence and absence of each item for the subjects. Further, the convergent validity of the GOHAI-T was assessed using Spearman’s rank correlation coefficient by examining the correlation between GOHAI-T scores and self-rated oral health for all subjects. The test-retest correlation coefficient for the GOHAI-T was assessed using Spearman’s rank correlation coefficient by re-interviewing 30 elderly subjects 1 week after the first interview. Cronbach’s alpha for internal consistency was 0.801 for the total scale and ranged between 0.764–0.813 for each item. The relation coefficient for the GOHAI-T score and self-rated oral health was 0.356 (p < 0.0001).This supported the convergent validity and showed that a low GOHAI-T score, indicating a higher level of impairment of OHRQoL, was significantly associated with poor self-rated oral health. The Spearman’s rank correlation coefficient for the test-retest was 0.797 (p < .001), indicating an acceptable level of reliability.

The internal consistency of the DSA (Cronbach’s alpha) was assessed in the presence and absence of each item for the subjects. Cronbach’s alpha was 0.887for the total scale and ranged between 0.857–0.881 for each item, indicating that the DSA had highly acceptable reliability. The convergent validity of the DSA was assessed using Spearman’s rank correlation coefficient by examining the correlation between total denture satisfaction scores and the general satisfaction of the sample subjects. The Spearman’s rank relation coefficient was 0.7583 (p < 0.001), which supported the convergent validity and showed that a high total denture satisfaction score was significantly associated with good general denture satisfaction.

Our data indicated that the skewness and kurtosis of GOHAI-T distribution were −0.77 and 3.25, respectively, and that these values were more normalized than the square-root-transformed GOHAI-T distribution (skewness = −1.02 and kurtosis = 3.89) or log-transformed GOHAI-T distribution (skewness = −1.07 and kurtosis = 4.81). Hence, the raw GOHAI-T scores were chosen for multiple linear regression analysis. Moreover, we conducted a robust linear regression to confirm the analysis results and found that all the statistically significant variables in the results of the robust regression were the same as those obtained using general multiple linear regression. Robust regression methods are designed such that they are not overly affected by violations of assumptions by the underlying data-generating process [33].

To assess the impacts of different blocks of predictors on OHRQoL, we conducted hierarchical multiple regression analysis using all independent predictors, with variance inflation factors under 5 to avoid multicollinearity in the whole model. The analysis was performed to determine the most predictive block of variables based on the GOHAI-T scores. The GOHAI-T score was used as a dependent variable and the blocks of independent variables were entered in the following steps: Step 1: socio-demographic characteristics, Step 2: dentition and denture status, Step 3: dental care service usage, Step 4: factors related to denture wearing. *R*^2^ shows the percentage of variability in the dependent variable that can be accounted for by all predictors. The change in *R*^2^ can be used to measure the amount of predictive power added to the model by the addition of another block of variables in the next step. We compared the standardized regression coefficients of independent variables to determine their impact on the GOHAI-T scores.

## Results

The basic characteristics of the study population are presented in Table 2. Data were collected from 277 subjects who wore removable dentures. More than half of them were women (52.7%), the mean age (± standard error) was 76.8 (±0.4) years, two-thirds had a level of education of high school or above (67.9%), the majority were living with others (81.2%), and more than three-fourths were able to afford their living expenses (78.7%). Further, the mean number of natural teeth was 7.8 ± 0.4. The distribution of the denture status was as follows: full-mouth complete denture, 24.9%; single complete denture, 24.9%; single partial denture, 24.6%; and full-mouth partial denture, 25.6%. Those who had regular dental checkups, treatment during toothache, and dental visits in the last year accounted for 22.4%, 10.8%, and 39.4% of the population, respectively. Regarding factors related to denture wearing, 24 (8.7%) subjects always/often presented with oral pain, 51 (18.4%) perceived that they had a loose denture, 28 (10.1%) had an oral ulcer, 21 (7.6%) had perceived halitosis, 31 (11.2%) had perceived dry mouth, and the total denture satisfaction score was 19.0 ± 0.3. In terms of the OHRQoL, the mean (± standard error) scores of the GOHAI-T subscales of PF, PSF, and PD were 47.8 ± 0.5, 14.3 ± 0.2, 20.8 ± 0.3, and 12.7 ± 0.1, respectively.

**Table 2:** Participants’ characteristics (*N* = 277)

Variables	n	%
**Socio-demographic characteristics**		
Gender		
Male	131	47.3
Female	146	52.7
Age (years)		
65–69	79	28.5
70–79	106	38.3
≥80	92	33.2
Mean ± SE (range)	76.8 ± 0.4 (65–90)	
Education level		
Less than high school	89	32.1
High school or above	188	67.9
Living status		
Alone	52	18.8
With others	225	81.2
Able to afford living expenses		
Yes	218	78.7
No	59	21.3
**Dentition and denture status**		
Remaining natural teeth		
Mean ± SE (range)	7.78 ± 0.4 (0–26)	
Denture status		
Full-mouth complete denture	69	24.9
Single complete denture	69	24.9
Single partial denture	68	24.6
Full-mouth partial denture	71	25.6
**Dental care service usage**		
Regular dental checkups	62	22.4
Treatment during toothache	30	10.8
Dental visits in the last year	109	39.4
**Factors related to denture wearing**		
Presence of oral pain	24	8.7
Perceived loose denture	51	18.4
Presence of oral ulcer	28	10.1
Perceived halitosis	21	7.6
Perceived dry mouth	31	11.2
Total denture satisfaction scores		
Mean ± SE (range)	19.0 ± 0.3 (6–30)	
**Oral health related quality of life**	Mean ± SE (range)	
GOHAI-T scores	47.8 ± 0.5 (22–60)	
Physical function	14.3 ± 0.2 (4–20)	
Psychosocial function	20.8 ± 0.3 (5–25)	
Pain and discomfort	12.7 ± 0.1 (3–15)	

Gender, education level, living status, ability to afford living expenses, denture status, regular dental checkups, treatment during toothache, and dental visits in the last year were not significantly associated with PF, PSF, PD, and total GOHAI-T scores. However, subjects who had oral pain, perceived that they had a loose denture, had an oral ulcer, and perceived that they had halitosis had lower PF and total GOHAI-T scores. Further, those who had perceived dry mouth had lower GOHAI-T scores (Table 3).

**Table 3:** **Oral health-related quality of life in denture-wearing elderly subjects (** ***N*** **= 277)**

Variables	Physical function	Psychosocial function	Pain and discomfort	GOHAI-T scores
	(Maximum score = 20)	(Maximum score = 25)	(Maximum score = 15)	(Maximum score = 60)
	Mean ± SE	Mean ± SE	Mean ± SE	Mean ± SE
Gender				
Male	14.37 ± 0.31	20.86 ± 0.36	12.84 ± 0.16	48.08 ± 0.67
Female	14.18 ± 0.32	20.66 ± 0.37	12.61 ± 0.19	47.44 ± 0.72
Education level				
Less than high school	14.16 ± 0.27	20.53 ± 0.31	12.63 ± 0.15	47.32 ± 0.60
High school or above	14.53 ± 0.37	21.26 ± 0.46	12.94 ± 0.23	48.73 ± 0.84
Living status				
Alone	14.01 ± 0.24	20.60 ± 0.29	12.68 ± 0.14	47.30 ± 0.55
With others	15.42 ± 0.49	21.46 ± 0.53	12.96 ± 0.30	49.85 ± 1.03
Able to afford living expenses				
Yes	14.39 ± 0.25	20.92 ± 0.28	12.89 ± 0.13	48.20 ± 0.53
No	13.88 ± 0.48	20.20 ± 0.63	12.14 ± 0.33	46.22 ± 1.18
Denture status				
Full-mouth complete denture	13.91 ± 0.41	20.67 ± 0.63	13.23 ± 0.23	47.81 ± 1.05
Single complete denture	13.29 ± 0.44	19.99 ± 0.54	12.26 ± 0.27	45.54 ± 1.00
Single partial denture	14.41 ± 0.48	21.06 ± 0.44	12.47 ± 0.26	47.94 ± 0.97
Full-mouth partial denture	15.46 ± 0.41	21.34 ± 0.42	12.96 ± 0.24	49.76 ± 0.85
Regular dental checkups				
No	14.04 ± 0.25	20.78 ± 0.30	12.73 ± 0.14	47.55 ± 0.56
Yes	15.10 ± 0.47	20.73 ± 0.51	12.73 ± 0.28	48.55 ± 1.04
Treatment during toothache				
No	14.35 ± 0.24	20.81 ± 0.26	12.69 ± 0.14	47.85 ± 0.52
Yes	13.67 ± 0.61	20.43 ± 0.98	13.07 ± 0.32	47.17 ± 1.47
Dental visits in the last year				
No	13.99 ± 0.30	20.34 ± 0.34	12.54 ± 0.16	46.88 ± 0.66
Yes	14.72 ± 0.32	21.42 ± 0.39	13.03 ± 0.20	49.17 ± 0.71
Presence of oral pain				
No	14.56 ± 0.23*	21.11 ± 0.24	12.88 ± 0.13	48.55 ± 0.48*
Yes	11.29 ± 0.57	17.13 ± 1.42	11.21 ± 0.53	39.63 ± 1.90
Perceived loose denture				
No	15.06 ± 0.22*	21.56 ± 0.22	12.97 ± 0.14	49.59 ± 0.45*
Yes	10.80 ± 0.41	17.24 ± 0.87	11.69 ± 0.29	39.73 ± 1.28
Presence of oral ulcer				
No	14.61 ± 0.23*	21.33 ± 0.22	12.94 ± 0.12	48.89 ± 0.46*
Yes	11.29 ± 0.61	15.75 ± 1.26	10.86 ± 0.46	37.89 ± 1.71
Perceived halitosis				
No	14.53 ± 0.22*	20.96 ± 0.26	12.82 ± 0.13	48.32 ± 0.49*
Yes	11.19 ± 0.74	18.33 ± 1.17	11.67 ± 0.60	41.19 ± 1.90
Perceived dry mouth				
No	14.46 ± 0.23	21.16 ± 0.24	12.83 ± 0.13	48.45 ± 0.47*
Yes	13.00 ± 0.69	17.91 ± 1.10	12.03 ± 0.42	42.94 ± 1.95

The p value was calculated by one-way analysis of variance for denture status and by two sample t-tests for the other factors.

*p < 0.05.

Denture satisfaction scores were positively associated with PF, PSF, PD, and GOHAI-T scores. We also found that the PF and PSF scores were significantly lower for subjects with a lower number of natural teeth. However, age was not significantly correlated with PF, PSF, PD, and total GOHAI-T scores (Table 4).

**Table 4:** **Correlations among oral health-related quality of life, age, and denture-related factors (** ***N*** **= 277)**

Variables	Physical function	Psychosocial function	Pain and discomfort	GOHAI-T score
	(Maximum score = 20)	(Maximum score = 25 )	(Maximum score = 15)	(Maximum score = 60)
	Coefficients	Coefficients	Coefficients	Coefficients
Age (years)	0.01	−0.05	0.09	0.01
Remaining natural teeth	0.17*	0.25*	0.06	0.03
Total denture satisfaction scores	0.65*	0.55*	0.56*	0.45*
Satisfaction with chewing ability	0.54*	0.49*	0.43*	0.35*
Satisfaction with ability to speak clearly	0.56*	0.47*	0.49*	0.38*
Satisfaction with appearance	0.43*	0.31*	0.43*	0.29*
Satisfaction with tasting ability	0.49*	0.44*	0.40*	0.31*
Satisfaction with retention of dentures (total,estimate)	0.50*	0.43*	0.40*	0.35*
Satisfaction with comfort (total, estimate)	0.52*	0.40*	0.47*	0.40 *

*p < 0.05.

Hierarchical multiple regression was performed to investigate the ability of factors related to denture wearing to predict GOHAI-T scores, after controlling for socio-demographic variables, dentition and denture status, and dental care service usage. Socio-demographic characteristics were entered in Model I and this model was not statistically significant, F (5, 271) = 1.99, p > 0.05. We added dentition and denture status to the socio-demographic characteristics in Model II, and the model as a whole explained a total of 12.4% of the variance, F (9, 267) = 4.20, p < .001. The education level, number of natural teeth, and denture status were predictors of GOHAI-T scores. In Model III, factors related to denture wearing were added, and the total variance explained by the model as a whole increased to 52.4%, F (15, 261) =19.13, p < 0.001. The introduction of factors related to denture wearing explained an additional 40.0%of the variance in GOHAI-T scores, Δ*R*^2^ = 0.40, F (6, 261) = 36.50, p < 0.001. Dental care service usage was added in Model IV, and the total variance explained by the model was 53.3%, F (18, 258) = 16.3, p < 0.001. The introduction of dental care service usage explained an additional 0.9% of the variance in GOHAI-T scores, Δ*R*^2^ = 0.009, F (3, 258) = 1.68, p > 0.05. In the final model, 6 of 18 predictor variables were statistically significant, with denture satisfaction having a higher beta value (β = 0.45, p < .001) than the remaining natural teeth (β = 0.26, p < .001), denture status, perceived loose denture, presence of oral ulcer, and educational level (Table 5).

**Table 5:** **Hierarchical multiple regression analysis of GOHAI-T scores**

Block and variables	Model I			Model II			Model III			Model IV		
	B	β	t	B	β	t	B	β	t	B	β	t
**Socio-demographic characteristics**												
Age	0.03	0.02	0.31	0.10	0.08	1.28	0.07	0.05	1.11	0.06	0.04	0.94
Gender												
Female vs. male	0.01	0.00	0.01	0.01	0.00	0.01	0.52	0.03	0.67	0.71	0.04	0.90
Education level												
Lower than high school vs. high school or above	1.51	0.09	1.33	2.56	0.15	2.29^*^	1.93	0.11	2.32^*^	1.92	0.11	2.28^*^
Living alone												
Yes vs. no	2.53	0.12	1.98^*^	2.26	0.11	1.82	1.73	0.08	1.87	1.55	0.07	1.66
Able to afford living expenses												
No vs. yes	−2.60	−0.13	−2.13^*^	−2.27	−0.11	−1.92	−1.26	−0.06	−1.43	−1.39	−0.07	−1.55
**Dentition and denture status**												
Remaining natural teeth				0.41	0.36	3.72^**^	0.24	0.21	2.87^**^	0.29	0.26	3.34^**^
Denture status												
Single CD vs. CD				−5.07	−0.27	−3.47^**^	−2.54	−0.14	−2.28^*^	−2.24	−0.12	−1.94
Single RPD vs. CD				−3.47	−0.18	−2.17^*^	−3.18	−0.17	−2.60^*^	−3.07	−0.16	−2.47^*^
Full-mouth RPD vs. CD				−4.43	−0.24	−2.04	−2.59	−0.14	−1.57	−2.52	−0.13	−1.52
**Factors related to denture wearing**												
Presence of oral pain (always/often)												
Yes vs. no							−1.28	−0.04	−0.89	−1.56	−0.05	−1.07
Perceived loose denture (always/often)												
Yes vs. no							−3.12	−0.15	−2.84^**^	−3.50	−0.17	−3.15^**^
Presence of oral ulcer (always/often)												
Yes vs. no							−4.83	−0.18	−3.46^**^	−4.23	−0.16	−2.97^**^
Perceived halitosis (always/often)												
Yes vs. no							−1.74	−0.06	−1.23	−1.90	−0.06	−1.35
Perceived dry mouth (always/often)												
Yes vs. no							−1.88	−0.08	−1.61	−2.00	−0.08	−1.71
Total denture satisfaction scores							0.77	0.44	8.79^**^	0.79	0.45	8.85^***^
**Dental care service usage**												
Regular dental checkups												
No vs. yes										−1.77	−0.09	−1.86
Treatment during toothache												
No vs. yes										1.52	0.06	1.16
Dental visits in the last year												
No vs. yes										−0.18	−0.01	−0.22
*R* ^2^			0.035			0.124^***^			0.524^***^			0.533^***^
Adjusted *R* ^2^			0.018			0.094^***^			0.496^***^			0.500^***^
*F*(df1, df2)			F(5,271) = 1.99			F(9,267) = 4.20			F(15,261) = 19.13			F(18,258) = 16.35
p			0.081			0.000			0.000			0.000
Δ*R* ^2^			0.035			0.089^***^			0.400^***^			0.009
*F* change			F(5,271) = 1.99			F(4,267) = 6.75			F(6,261) = 36.50			F(3,258) = 1.68
p for *F* change			0.081			0.000			0.0000			0.172

Hierarchical multiple regression for the association between GOHAI-T scores and all block variables with VIF set at < 5 to avoid multicollinearity.

Δ*R* ^2^: change in*R* ^2^; statistical significance: ^*^p < 0.05, ^**^p < 0.01, ^***^p < 0.001.

Model I: Block1 (socio-demographic variables).

Model II: Block1 (socio-demographic variables) + block 2 (dentition and denture status).

Model III: Block1 (socio-demographic variables) + block 2 (dentition and denture status) + block 3 (factors related to denture wearing).

Model IV: Block1 (socio-demographic variables) + block 2 (dentition and denture status) + block 3 (factors related to denture wearing) + block 4 (dental care service usage).

## Discussion

Despite the important findings, our study had several limitations that should be considered with some caution when interpreting the results. First, we did not use a population-based sample, but a convenience sample from the Senior Citizens’ College of Kaohsiung City, and thus, our study represents only a subgroup of the public. Future research should focus on generalizing the findings to the elderly Taiwanese people wearing removable dentures. Second, the present study is characterized by a cross-sectional design, and therefore, we cannot make inferences with respect to the direction of the observed associations. The third limitation was related to the study instrument. Because no a Chinese version of the GOHAI for Taiwanese had been validated when the study was performed, the original English version was translated by the researchers and was examined among a convenience sample of elderly subjects in Kaohsiung City for its construct validity and internal reliability. Therefore, further studies may be required to determine whether this version can be applied to the elderly Taiwanese population.

To enable the development of patient-oriented approaches in public health care and provide appropriate oral health care to elderly people wearing removable dentures, it is important to know which predictors actually affect the OHRQoL of elderly people. Therefore, the present study aimed to assess the factors of OHRQoL among elderly people wearing removable denture. Results of the hierarchical multiple regression showed that factors related to denture wearing were most strongly associated with OHRQoL, with denture satisfaction being the strongest predictor of OHRQoL, and the number of remaining natural teeth was the second highest predictor. In addition, the OHRQoL of elderly people wearing removable dentures could also be predicted by education level, denture status, perceived loose denture, and presence of oral ulcer. Thus, among the predictors analyzed in this study, denture satisfaction was the best predictor of OHRQoL of elderly people wearing removable dentures.

The findings of this study suggested that high satisfaction with removable dentures was significantly related to a high GOHAI-T score, indicating a low level of impairment of OHRQoL. This is in line with the findings of previous studies that showed a positive correlation between self-reported denture satisfaction and OHRQoL in the elderly [23]. In their 2-year longitudinal analysis, Stober et al. [19] showed that elderly patients’ satisfaction with complete dentures was associated with OHRQoL, based on scores on the shortened version of the OHIP. Recently, Kuo et al. [7] reported that patients’ satisfaction with complete dentures was significantly related to an improvement in their OHRQoL, as measured using the OHIP-14. In their cross-sectional analysis, Lee et al. [10] showed that if the elderly are satisfied with their dentures, OHRQoL, assessed using the OHIP, may be unaffected by oral health problems.

A recent study described a positive correlation between professional ratings of removable denture quality and a low OHIP score, indicating a low level of impairment of OHRQoL [29]. However, a poorly fitting denture may be well tolerated in one person, while a well fitting denture may create problems for another [30]. Garrett et al. [34] reported that 55% of 21 patients with poorly fitting denture [31] had moderate-to-complete denture satisfaction, which was found by the examining dentists assessing need for denture replacement. Närhi et al. [35] found a weak or statistically nonsignificant correlation between patients’ denture satisfaction and clinical measurements such as anatomic conditions, as well as denture quality. Thus, denture satisfaction is not based on the technical quality of the dentures alone. Moreover, Turker et al. [30] suggested that although patients seek technical advice, psychological and emotional factors play an important role in their poor adaptation to the denture. Another study showed that the dentist–patient relationship and psychological factors had a great impact on patients’ acceptance of and adaptation to removable dentures [33]. Therefore, in addition to clinical and technical skills, gaining a better understanding of patient behavior and psychology, and improving communication are crucial to improving patients’ denture satisfaction [30, 33].

In the present study, subjects with a perceived loose denture gained significantly lower mean GOHAI-T scores, indicating a worse OHRQoL than those without a perceived loose denture. This result is in accordance with those of previous studies [23] showing that denture loosening was a significant contributing factor to low OHRQoL. Moreover, Hassel et al. [11] showed that improving retention of previously insufficiently retained dentures was positively associated with OHRQoL, as measured using the OHIP-49 (German version), among removable denture wearers. Hassel et al. further indicated that denture loosening affects many aspects of quality of life, both psychological and social. Komagamine et al. [22] also reported that sufficient retention of lower dentures is important to improving OHRQoL in edentulous patients. Hence, it is necessary to identify and improving retention of previously insufficiently retained dentures in order to improve the OHRQoL of elderly persons wearing removable dentures.

Removable dentures can injure oral tissues, and their use is associated with a high frequency of oral mucosal lesions [36]. In the present study, subjects who had had an oral ulcer were more likely to report a poorer OHRQoL than their counterparts without oral ulcer experiences. This finding is consistent with the results of many previous studies showing that oral ulcers were associated with a poor OHRQoL [37]. In a study evaluating the sensitivity of patient-centered outcome measures to treatment, McGrath et al. [34] suggested that patients with ulcers and symptomatic oral mucosal lesions had poorer OHRQoL (measured using the OHIP-14) than their counterparts with non-symptomatic lesions. Recently, Suliman et al. [13] showed that oral ulcerative lesions were significantly associated with oral impacts on daily performance, indicating poor quality of life. Moreover, Mandali et al. [38] suggested that recall visits and shortened denture usage are essential for prevention of oral mucosal lesions. Therefore, to improve OHRQoL of elderly population, it is important to educate and regularly review patients who wear dentures with regard to oral tissue injuries.

Our results demonstrated that the number of remaining natural teeth was positively associated with OHRQoL; thus, the larger the number of remaining natural teeth, the lower the impact on the OHRQoL. This result was in accordance with those of many previous studies [12, 16]. Zaitsu et al. [17] suggested that the number of missing teeth was significantly associated with low GOHAI scores, indicating a poor OHRQoL. In their meta-analysis, Gerritsen et al. [18] showed that tooth loss is associated with impairment of OHRQoL, and this association seems to be independent of the OHRQoL assessment instrument used and the context of the included samples. Moreover, Jain et al. [12] indicated that a decrease in the number of remaining natural teeth was correlated with poor OHRQoL, independent of the effect of age and gender. Therefore, they concluded that all populations with complete natural dentition show a good OHRQoL.

Our study also showed that denture status was a predictor of OHRQoL, as measured by the GOHAI-T, and that removable partial denture (RPD) wearers experienced a more adverse impact of the dentures on their OHRQoL than did complete denture wearers. Another study found that RPD users experienced impaired OHRQoL more often than did CD users, based on responses to the OHIP [39]. In addition, Wong et al. [21] reported that in a sample of community-dwelling elderly Chinese people, those who wore RPD experienced a greater OHRQoL impairment (measured by the GOHAI) than did CD wearers. These findings probably result from the unrealistic expectations of the wearers from their dentures, i.e., RPD wearers tend to compare their dentures with natural teeth. In contrast, researchers have shown that CD wearers have usually previously used RPD and might accept the lack of teeth and oral discomfort as a part of the aging process. Thus, CD wearers might be more accepting of the limitations of denture functions than RPD wearers are [22, 39].

In our study, educational level had a significant influence on GOHAI-T scores. Subjects with a higher education level were more likely to have poorer OHRQoL, indicating more perceived dental problems in this group than in those with an education level lower than high school. A possible explanation for this trend may be related to a person’s expectations. Subjects with a lower level of education may have lower standards or expectations in their evaluation of health satisfaction or life satisfaction and, accordingly, be more likely to be satisfied than those who are more highly educated [40]. Another possible reason is that subjects with a higher education level may be more concerned about problems with oral health and esthetics than those with a lower education level are, as this may be less accepted in the social circles of those who are more highly education. Therefore, those with a lower education level and lower expectations may report higher GOHAI-T scores, indicating a better OHRQoL.

Compared to other international literature dealing with OHRQoL in the elderly, we found lower GOHAI-T values in this study. One possible reason for this is that the subjects in this study were elderly individuals wearing removable dentures. Hogenius et al. [8] showed that compared to people with natural teeth, those who wear removable dentures experience more significant negative impacts on social and emotional aspects of life. Another possible reason may be that the subjects in this study were sourced from the Government Senior Citizens’ Service Center in Taiwan. Elderly people attending this center tend to have a higher education level and better socioeconomic status. Subjects with a higher level of education may have higher expectations relating to their oral health satisfaction and, accordingly, be more likely to have lower GOHAI-T values [15].

The present study was performed in 2009, at which time the GOHAI had not yet been translated for use in Taiwan. A Chinese version of the GOHAI for older people living in Hong Kong and southern China was developed in 2002 [41]. Though the Taiwanese language is influenced by Chinese culture, there are a number of differences between the spoken and written Chinese of Hong Kong and that of Taiwan, so, the wording of the questions in the Chinese version of the GOHAI could confuse elderly Taiwanese individuals. Therefore, in the current study, we did not use the Chinese version of the GOHAI for assessing OHRQoL measures. Instead, a Chinese version of the GOHAI for use with Taiwanese people specifically (GOHAI-T) was developed.

A strength of the current study was that we simultaneously controlled both for factors related to denture wearing and for relevant influencing factors mentioned in previous similar studies. In the final model, the overall explanatory power was 0.533. The present findings confirmed that factors related to denture wearing were most strongly associated with OHRQoL, with denture satisfaction being the strongest predictor. Therefore, in clinical praxis, denture satisfaction is a useful tool to evaluate the effect of denture treatment on OHRQoL. However, a weakness of the study was that the nature of the causality in which denture satisfaction is used to predict OHRQoL was still unclear due to the cross-sectional design. Therefore, OHRQoL could not be measured by the total denture satisfaction score instead of the GOHAI-T score.

## Conclusions

In conclusion, of the factors analyzed in this study, those related to denture wearing were most strongly associated with OHRQoL, and denture satisfaction was the strongest predictor among them. It is suggested that denture satisfaction is useful as a tool for assessing the effect of denture treatment on the OHRQoL of elderly individuals wearing removable dentures.

## Abbreviations

OHRQoL: Oral health-related quality of life
OHIP: Oral health impact profile
DSA: Denture-satisfaction assessment
PD: Pain or discomfort
PF: Physical function
PSF: Psychosocial function
NT: Natural teeth
GOHAI: Geriatric oral health assessment index.
